# Twelve-Month Results from a Prospective Clinical Study Evaluating the Efficacy and Safety of Cellular Bone Allograft in Subjects Undergoing Lumbar Spinal Fusion

**DOI:** 10.3390/neurolint14040070

**Published:** 2022-10-26

**Authors:** Joshua Wind, Daniel Park, Todd Lansford, Pierce Nunley, Timothy Peppers, Anthony Russo, Hamid Hassanzadeh, Jonathan N. Sembrano, Jung Yoo, Jonathan Sales

**Affiliations:** 1 Sibley Memorial Hospital, 5255 Loughboro Rd. NW, Washington, DC 20016, USA; 2 Beaumont Hospital, 3601 W 13 Mile Rd., Royal Oak, MI 48073, USA; 3 Trident Medical Center, 9330 Medical Plaza Dr., North Charleston, SC 29406, USA; 4 Spine Institute of Lousiana, 1500 Line Ave., Shreveport, LA 71101, USA; 5 Scripps Memorial Hospital, 9888 Genesee Ave., San Diego, CA 92037, USA; 6 Montana Orthopedics, 435 S Crystal St. 400, Butte, MT 59701, USA; 7 John Hopkins Medicine, 6420 Rockledge Dr., Bethesda, MD 20817, USA; 8 Department of Orthopedic Surgery, University of Minnesota, 909 Fulton St SE, Minneapolis, MN 55455, USA; 9 OHSU Hospital, 3303 S Bond Ave, Portland, OR 97239, USA; 10 Summit Spine, 9155 SW Barnes Rd. 210, Portland, OR 97225, USA

**Keywords:** lumbar fusion, arthrodesis, cellular allograft, Trinity Elite

## Abstract

Background: While autologous bone grafts remain the gold standard for spinal fusion procedures, harvesting autologous bone is associated with significant complications, including donor site infection, hematomas, increased operative time, and prolonged pain. Cellular bone allograft (CBA) presents an alternative to autologous bone harvesting, with a favorable efficacy and safety profile. The current study further investigates CBA as an adjunct to lumbar spinal fusion procedures. Methods: A prospective, multicenter, open-label clinical study was conducted in subjects undergoing lumbar spinal fusion with CBA (NCT 02969616). Radiographic fusion status was assessed by an independent review of dynamic radiographs and CT scans. Clinical outcome measures included the Oswestry Disability Index (ODI) and visual analogue scale (VAS) for back and leg pain. Adverse-event reporting was conducted throughout 12 months of follow-up. Available subject data at 12 months were analyzed. Results: A total of 274 subjects were enrolled into the study, with available data from 201 subjects (73.3%) who completed 12 months of postoperative radiographic and clinical evaluation at the time of analysis. Subjects had a mean age of 60.2 ± 11.5 years. A higher number of women (n = 124, 61.7%) than men (n = 77, 38.3%) were enrolled, with a collective mean BMI of 30.6 + 6.5 kg/m^2^ (range 18.0–51.4). At month 12, successful fusion was achieved in 90.5% of subjects. A significant (*p* < 0.001) improvement in ODI, VAS-back, and VAS-leg clinical outcomes was also observed compared to baseline scores. One adverse event related to CBA (postoperative radiculopathy) was reported, with surgical exploration demonstrating interbody extrusion of graft material. This subject reported successful fusion at month 12. Conclusions: CBA represents a viable substitute for harvesting of autograft alone with a high rate of successful fusion and significant improvements in subject-reported outcomes, such as pain and disability. Positive benefit was observed in subjects reporting single and multiple risk factors for pseudoarthrosis.

## 1. Introduction

Lumbar spinal fusion is frequently utilized to treat a variety of degenerative, traumatic, and oncological spine disorders with the overarching goal of improving spinal instability or weakness, to reduce pain, or correct deformities (e.g., scoliosis). Spinal fusion techniques mimic the normal healing process of the bone by using bone or biological and synthetic bone-like materials to facilitate permanent connection between spinal vertebrae. The primary sources for bone graft material include autologous (collected from the patient’s own body), allograft (collected from cadaveric bone), and synthetic substitutes. Traditionally, fusion is achieved by mechanical roughening and decortication of the bone surface followed by packing of the joint space with the selected bone graft material. The selection of the bone graft material is critical for successful fusion outcomes [1].

Spinal fusion success is dictated by three distinct properties: (1) an osteoconductive scaffolding for support, (2) osteoinductive molecular signaling for promotion, and (3) osteogenic cells to facilitate fusion [1,2]. Autologous iliac crest bone graft (ICBG), which provides all three elements, has remained the gold standard for spinal fusion bone grafting. The iliac crest (located at the top of the pelvis) supplies a relatively large volume of bone that can be harvested for spinal fusion procedures [3]. The morbidity of iliac crest grafting is substantial, and complications are well documented, including increased bleeding, fracture, pseudoaneurysm of the pelvic vasculature, arteriovenous fistula, hernia, neurological injury, and pain [4,5,6]. As high as 38% of procedures show donor site morbidities, highlighting the impact of these complications [7,8,9,10,11,12,13].

Locally harvested autograft bone can be used during an arthrodesis instead of the iliac crest; however, supply limitation is also a factor [3]. Alternative modalities to autograft exist and include bone marrow aspirate, bone allografts, synthetic bone void fillers, and bone morphogenetic protein (BMP) [12,14,15]. These alternative modalities provide one or two of the necessary elements for successful fusion, but do not target all three osteoconductive, osteoinductive, and osteogenic properties present in autograft bone.

Cellular bone allograft (CBA) represents a relatively new addition to allograft technologies. CBA is designed to maintain the viable osteogenic cells within an osteoconductive corticocancellous bone matrix in addition to demineralized bone to enhance osteoinductivity [16,17]. CBA contributes all three of the critical elements necessary for successful bone formation, but more importantly without the donor site morbidity associated with bone graft harvesting. Therefore, CBA may provide similar benefits as a bone graft source to autologous bone grafts while minimizing their limitations.

Despite the widespread use of CBA in lumbar and cervical spinal fusion surgery, there is limited clinical evidence detailing associated patient outcomes [18,19,20,21]. The current study further investigates the safety and efficacy of CBA. A prospective, multicenter clinical trial in subjects undergoing elective single- or multilevel lumbar arthrodesis for degenerative conditions was conducted. Clinical and radiographic outcomes at 12 months of follow-up are presented in the current report, while subjects continued to be assessed for clinical and radiographic outcomes through 24 months.

## 2. Materials and Methods

### 2.1. Subjects

The study was conducted in accordance with Good Clinical Practice Guidelines and approved by associated ethical review boards in accordance with the Declaration of Helsinki. The study was registered through clinicaltrials.gov, identifier NCT 02969616. Subjects were enrolled only following informed consent. Adult subjects (≥18 years) that had failed at least 6 months of conservative care who planned to undergo posterolateral fusion (1–4 levels) or interbody fusion (1–2 levels) and met the predefined inclusion/exclusion criteria were enrolled. Subjects who had had prior lumbar spine fusion surgery at a level currently scheduled for surgery, were undergoing treatment for malignancy or had undergone treatment for malignancy within the last 5 years (benign skin cancer permitted), an active local or systemic infection, or were undergoing adjunctive treatment for local or systemic infection were excluded.

### 2.2. Study Design and Treatment

The current study employed a prospective, postmarket, multicenter, open-label, clinical study design to evaluate the efficacy and safety of CBA. Subjects were prospectively enrolled at 9 clinical sites across the United States and screened for inclusion/exclusion criteria. The surgical approach and technique and placement/location of the bone graft were determined at the discretion of the treating surgeon. Subjects received CBA using the Trinity Elite matrix (Trinity Elite, MTF Biologics, Edison, NJ, USA), a novel, allogeneic, cancellous bone matrix containing viable osteogenic cells and a demineralized cortical bone component. Trinity Elite was used as the primary (>50% by volume) bone graft substance, with augmentation of up to 50% of locally harvested autograft and/or cancellous allograft chips. No additional bone graft substitutes were allowed.

### 2.3. Assessments

This analysis included all data available up to the 12-month time point. Subjects continued to be followed up to 24 months (NCT 02969616) as dictated in the full protocol. Radiographic fusion was assessed at 12 months by an independent review (TELOS Partners, Warsaw, IN, USA and MMI, Houston, TX, USA). Successful fusion was defined as (1) lack of angular and translational motion (<3 deg and <3 mm, respectively) on quantitative motion analysis (QMA) and (2) the presence of bridging bone across the adjacent endplates or transverse processes on thin-cut CT scans. Both fusion criteria had to be met for the subject to be considered a fusion success. Subjects undergoing multilevel procedures had to demonstrate fusion success at all treated levels to be considered a fusion success. Dynamic X-rays (flexion/extension) for QMA were obtained at 3 months, 6 months, and 12 months postoperatively, while CT scans were obtained at 12 months. Clinical outcomes included the Oswestry Disability Index (ODI) and visual analogue scale (VAS) for back and leg pain. Clinical outcomes were obtained at baseline, 6 weeks, 3 months, 6 months, and 12 months postoperatively. Adverse events were recorded from surgery through 12 months postoperatively for each subject, including the event’s relatedness, severity, and outcome.

### 2.4. Statistical Analysis

Data were analyzed with SASS version 9.4. Counts and percentages are reported for categorical baseline variables. Mean, standard deviation (SD) and range are reported for continuous variables. Preoperative and postoperative subject-reported outcomes were compared with a paired-sample *t*-test. Alpha was set at 0.05 and a *p*-value < 0.05 was considered significant.

## 3. Results

### 3.1. Subject Demographics and Surgical Procedure

Out of the 274 subjects enrolled, prior to the 12-month follow-up visit, nine subjects withdrew their informed consent, four subjects were withdrawn by a treating surgeon, and there was one death unrelated to the study treatment. Of the 260 subjects available for the 12-month follow-up, 201 subjects (77.3%) had completed 12 months of postoperative radiographic and clinical evaluation at the time of this analysis. The study cohort had a mean age of 60.2 + 11.5 (range 28–82) years, included 124 (61.7%) women, and a mean BMI of 30.6 + 6.5 kg/m^2^ (range 18.0–51.4). Thirty-one subjects were current smokers (15%), 41 were diabetic (20%), and 15 were osteoporotic (8%) (Table 1).

Sixty subjects (30%) underwent multilevel lumbar arthrodesis. Forty-nine subjects (24%) underwent anterior lumbar interbody fusion (ALIF), 53 (26%) underwent lateral or oblique lateral lumbar interbody fusion (LLIF, DLIF, XLIF or OLIF), 86 (43%) underwent posterior interbody fusion (PLIF or TLIF), and 13 (6%) underwent posterolateral lumbar fusion (PLF) (Table 2).

### 3.2. Radiographic Fusion Outcomes

**Figure 1:**
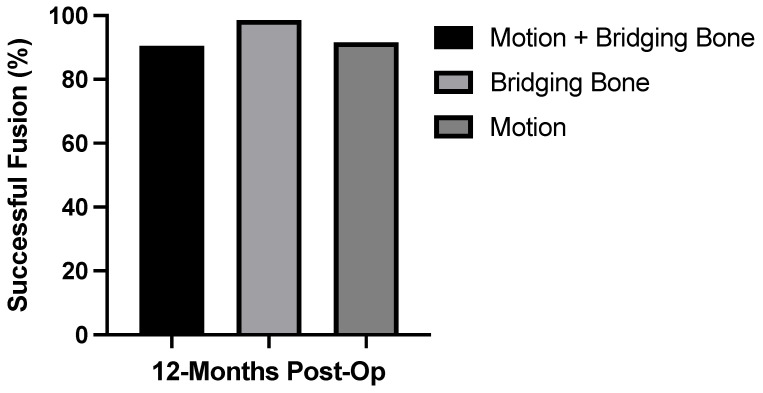
Spinal fusion success rate. A high rate of fusion success was observed in lack of motion + bridging bone, bridging bone only, and lack of motion only.

**Figure 2:**
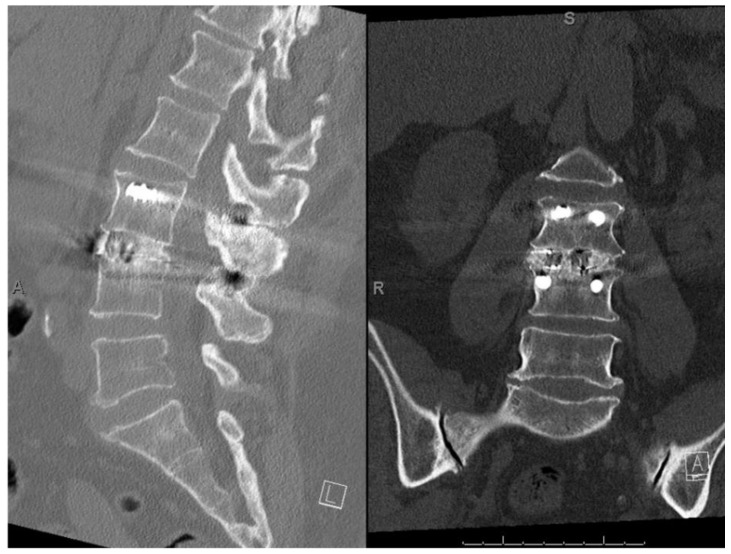
Spinal fusion success. One-year postoperative CT scan demonstrating successful bridging bone on sagittal and coronal reconstructions.

Fusion success (both lack of angular/translational motion and the presence of bridging bone) was confirmed in 182 of 201 subjects (90.5%) (Figure 1). Bridging bone was observed in 198 of 201 subjects (98.6%). Less than 3° angular motion and <3 mm translation was observed in 184 of 201 subjects (91.6%). Nineteen of the 201 subjects (9.5%) had evidence of pseudoarthrosis; however, no revision surgeries were reported. Figure 2 shows a representative CT scan of successful spinal fusion.

Fusion success rates by the number of levels treated and surgical approach are presented in Table 2. There were 141 subjects (70%) that underwent one-level arthrodesis, 57 (28%) underwent two-level arthrodesis, 1 underwent three-level arthrodesis, and 2 underwent four-level arthrodesis. The rate of fusion success was high among all surgical approaches.

Fusion rates for subjects with risk factors that increase the potential for nonunion are presented in Table 3. The rate of successful fusion for subjects with no risk factors, 85.1% (n = 27), did not differ significantly from those with multiple risk factors, 90.5% (n = 106; *p* = 0.497). No individual risk factor had a statistically significant association with pseudoarthrosis. Osteoporotic subjects had the lowest rates of fusion (80%); however, there were only 15 subjects with this risk factor.

### 3.3. Clinical Outcomes

**Figure 3:**
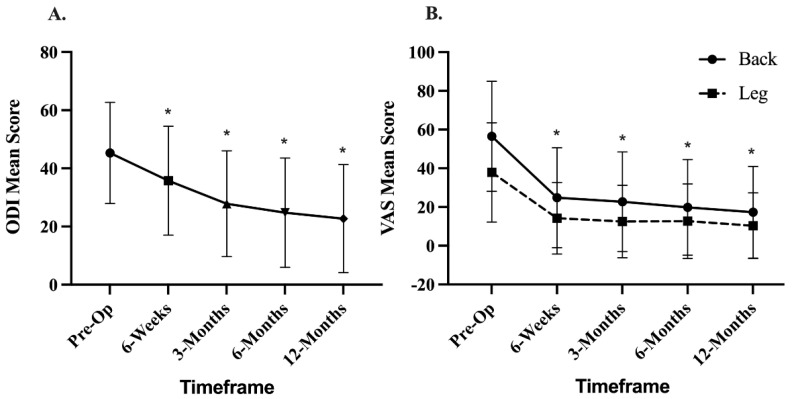
ODI and VAS scores through 12 months postoperation. (**A**). Mean ODI scores up to 12 months. (**B**). Mean VAS-back and VAS-leg scores up to 12 months. A significant improvement in ODI and VAS score was observed at all time points through 12 months, * *p* < 0.0001. ODI, Oswestry Disability Index; VAS, visual analogue scale.

Clinical outcomes are presented in Figure 3. Mean preoperative ODI score was 44.92 ± 17.11 and improved to 21.89 ± 18.51 (*p* < 0.001) at 12 months. Mean preoperative VAS-back score was 56.5 ± 28.4 and improved to 17.3 + 23.6 (*p* < 0.0001) at 12 months. Mean preoperative VAS-leg score was 37.90 ± 25.60 and improved to 10.38 ± 16.95 (*p* < 0.0001) at 12 months. Subjects that did not achieve fusion success (n = 19) still reported improvements in VAS-back from baseline (50.0 ± 34.2) to 12 months (13.0 ± 16.4; *p* = 0.0002), and VAS-leg scores from baseline (33.71 ± 31.80) to 12 months (7.16 ± 12.43; *p* = 0.0011). Similarly, improvements in disability (ODI) were also reported from baseline (41.5 ± 14.1) to 12 months (14.4 ± 16.6; *p* = 0.0001).

### 3.4. Adverse Events

Adverse events were characterized by relatedness to the bone graft and/or procedure and event severity. Only one adverse event related to CBA was reported as postoperative radiculopathy, with surgical exploration demonstrating extrusion of graft material from the interbody. This subject reported successful fusion at 12 months.

## 4. Discussion

A rise in the prevalence of lumbar spinal fusion procedures has highlighted procedural complications and refocused efforts towards improving fusion success with alternative strategies [22,23]. The traditional approach using autologous ICBG is associated with significant morbidities [7,8,9,10,11,12]. Therefore, exploration into other procedural options is of high interest. Various clinical and demographic risk factors impact on the rate of pseudoarthrosis; however, selection of graft material is a modifiable factor the surgeon can control. The current study explored the impact of CBA for lumbar spinal fusion and found a high rate of fusion success (90.5%), as assessed by dynamic radiographs and CT, and positive effects on clinical outcomes.

Reported rates for fusion success vary across studies depending on surgical approach and source of bone graft substitute. Lumbar fusion for degenerative indications is associated with the greatest measured practice variation of any surgical procedure [24]. The reported fusion rate for procedures with local bone graft materials is 65–93% [25,26,27,28,29,30]. A recent meta-analysis (2020) showed a 62.6% fusion success rate [29]. Similarly, a comprehensive 2017 meta-analysis found that fusion success when using autograft ranged from 58–68% [15]. Fusion rates in patients undergoing TLIF were reported to be 94.5% and 93.0% in a prospective randomized study using ICBG and local autograft, respectively [31].

Current evidence supports high rates of fusion success using CBA and provides the underlying rationale for further exploration into this alternate modality as a beneficial source for bone graft in lumbar and cervical spine fusion. These allografts have all three principal components of a desirable bone graft substitute: osteoconductivity, osteoinductivity, and osteogenicity. Peppers and Vanichkachorn reported high fusion rates (93–94%) using an alternate CBA, Trinity Evolution (MTF Biologics, Edison, NJ, USA), as an adjunct to fusion in patients undergoing single and two-level ACDF at 12 months. It is noteworthy that this patient population was inclusive of those patients with single and/or multiple risk factors to bone healing [32,33]. Musante et al. reported 90% fusion success rates in patients undergoing PLF, with no significant difference in fusion rates among patients with and without risk factors to fusion [34]. In addition, Vivigen CBA (Depuy-Synthes, Raynham, MA, USA) has demonstrated high spinal fusion rates in multiple studies [35]. Multilevel posterolateral fusion with ViviGen demonstrated a fusion rate of 98.7% (graded via radiographs only) [21]. A subsequent study conducted by Elgafy et al. showed a fusion rate of 91.7% using ViviGen when graded by radiographs and CT [20]. Ammerman et. al. reported a fusion rate of 91.3% in a small cohort (n = 23) of minimally invasive TLIF cases that used Osteocel (NuVasive, San Diego, CA, USA) at 12 months follow-up [18]. Similarly, Tohmeh et al. reported a fusion rate of 90.2% using Osteocel in a cohort of patients (n = 40) undergoing XLIF procedures at 12 months [19]. The fusion rate of 90.5% reported in the current study compares favorably with existing literature on lumbar spine fusion.

An improvement in other important clinical outcomes, including ODI, VAS for back and leg, and rate of adverse events, was also observed in the current study. Copay et al. defined a meaningful clinically important difference (MCID) of 12.8 for ODI, and 1.2 for VAS-back, and 1.6 for VAS-leg [36]. The average improvement in this study at 12 months was 23 for ODI, 3.9 for VAS-back, and 5.5 for VAS-leg, and all exceeded the established MCID. Positive benefits in these outcome measures are of significance when interpreting the quality of life for the patient postsurgery in regard to associated morbidity.

Although some studies have shown fusion success and that clinical outcomes are affected by risk factors, such as elevated BMI (>25), diabetes, older age, osteoporosis, and smoking [36,37,38], our results show no significant impact on success. However, given the low number of study participants, more data are needed across a broad spectrum of patients and risk factors. The extension of this study provides additional efficacy and safety data out to 24 months and will be available in a subsequent publication.

These study findings provide additional support for the viability and efficacy of CBA in spinal fusion. CBAs provide a unique alternative to autograft, given that they preserve the inherent properties of osteoinductive and osteogenic components retained within the bone matrix. However, due to distinct propriety processing techniques, one CBA cannot be easily compared to another without a standardized system. Ultimately, fusion rates using CBA in this study were comparable to ICBG fusion rates reported in the literature. These favorable fusion rates are consistent across the entire subject population, regardless of risk factors for pseudoarthrosis. This use of CBA eliminates donor site morbidity and complications associated with BMP products, namely, heterotopic bone, seroma formation, and radiculitis [37,38,39].

While positive findings were observed on clinical outcomes and fusion rate success, limitations to this study do exist. The analysis included subjects that underwent different surgical procedures. Study findings thus present real-world evidence of varied lumbar fusion procedures observed within the patient population. The study design did not account for a controlled comparative arm. However, review of the literature demonstrates a fusion rate and improvements in pain and disability in this study that are comparable to other studies. Regardless of these limitations, the findings from this study furthers evidence for the utility of CBA bone graft substitutes for lumbar spinal fusion.

## 5. Conclusions

The current study investigated the impact of CBA as an alternate source for bone grafting material in spinal fusion procedures. A successful fusion rate of 90.5% was observed at 12 months postoperatively in subjects who received posterolateral fusion (1–4 levels) or interbody fusion (1–2 levels) procedures. Improvements in clinical outcome measures, including ODI and VAS scores, were also observed. Altogether, these study findings provide additional support for the efficacy and safety of CBA in spinal fusion procedures.

## Figures and Tables

**Table 1:** Baseline Demographics and Clinical Characteristics.

Characteristic	Subjects (N = 201)
Age (years), Mean (Range)	60.2 ± 11.5 (28–82)
Female; Male, n (%)	124 (61.7); 77 (38.3)
BMI, Mean (Range)	30.6 ± 6.5 kg/m^2^ (18.0–51.4)
Smoker, n (%)	31 (15.4)
Diabetes, n (%)	41 (20.4)
Osteoporosis, n (%)	15 (7.5)

**Table 2:** Fusion Success at 12 months by Level Treated and Surgical Approach.

	Total Subjects N	Fusion Success N (%)
Level		
1 Level	141	128 (90.8)
2 Level	57	52 (91.2)
3 Level	1	1 (100.0)
4 Level	2	1 (50.0)
Surgical Approach		
Anterior Lumbar Interbody Fusion (ALIF)	49	40 (81.6)
Posterior Lumbar Interbody Fusion (TLIF/PLIF)	86	80 (93.0)
Lateral or Oblique Lateral Lumbar Interbody Fusion (OLIF/XLIF/LLIF/DLIF)	53	50 (94.3)
Posterolateral Lumbar Fusion (PLF)	13	12 (92.3)

**Table 3:** Fusion Success Rate by Risk Factor.

Risk Factor	Fusion Success	*p*-Value
BMI ≥ 30	93.4% (n = 107)	0.1324
Smoking	87. 1% (n = 31)	0.5036
Age + 65	88.1% (n = 84)	0.3140
Diabetes	85.3% (n = 41)	0.2037
Osteoporosis	80.0% (n = 15)	0.1567
Multiple Risk Factors	90.5% (n = 106)	0.4970

## Data Availability

Data supporting the findings of this study are available on request.
